# Biodegradability of Bioplastics in Managed and Unmanaged Environments: A Comprehensive Review

**DOI:** 10.3390/ma18102382

**Published:** 2025-05-20

**Authors:** Giovanni Gadaleta, Johana Carolina Andrade-Chapal, Sara López-Ibáñez, María Mozo-Toledo, Ángela Navarro-Calderón

**Affiliations:** Biodegradability & Compostability Laboratory, AIMPLAS—Plastics Technology Centre, C. Gustave Eiffel n. 4, 46980 Paterna, Valencia, Spain; jandrade@aimplas.es (J.C.A.-C.); salopez@aimplas.es (S.L.-I.);

**Keywords:** biodegradation, bioplastics, compostability, certification, industrial composting, soil biodegradation, water biodegradation

## Abstract

The production and utilization of conventional plastics have raised concerns regarding plastic waste management and environmental safety. In response, the emergence of biodegradable bioplastics presents a possible solution for sustainability. On the other hand, the efficacy of biodegradation is strictly dependent on both the bioplastic type and the conditions in which the biodegradation occurs. This review offers a comprehensive overview of the biodegradation behavior of several bioplastics under a managed (industrialized or controlled) environment, such as industrial composting and anaerobic digestion (at either mesophilic or thermophilic temperature), as well as under less studied unmanaged (natural or open) environments, including soil, seawater, and freshwater. Although the biodegradation trend of some bioplastics is well known, further investigation should be pursued for others in order to clearly have the knowledge and the ability to choose the most viable bioplastic for a specific application and future end-of-life.

## 1. Introduction

Plastic has been considered the most relevant material during this last century, playing a fundamental role in modern life due to its convenience and versatility. However, the advantages of plastic materials have led to an ongoing increase in their use and production, which has affected—and continues to affect—the environment [1]. The widespread use and mismanagement of plastic products have contributed to environmental, economic, and social issues [2]. As society grapples with the consequences of plastic consumption, the need for sustainable alternatives has never been more urgent.

Bioplastics have been proposed as one of the possible alternatives to conventional plastics, with the potential to mitigate the environmental footprint of plastic production and disposal, offering a pathway towards a more circular and sustainable economy. According to European Bioplastics, bioplastics are materials that are either bio-based or biodegradable, or possess both properties [3]. A bio-based material or product is (fully or partially) obtained from natural biomass, while a biodegradable material or product could be converted into natural substances during a biological process by microorganisms, fungi, and bacteria. The presence of microbial activity differentiates biodegradation from other types of chemical–physical degradation, such as photo-degradation, oxo-degradation, etc.

Focusing on biodegradable bioplastics, several types are currently on the market, and new materials are also being developed and produced, such as polylactic acid (PLA), polyhydroxyalkanoates (PHAs), poly(butylenesuccinate) (PBS), polycaprolactone (PCL), poly(butylene adipate-co-terephthalate) (PBAT), and starch- and cellulose-based bioplastics.

PLA is a biodegradable and bio-based polymer derived from renewable resources such as corn starch or sugarcane. Due to its properties and processability, PLA is one of the most commonly used bioplastics, with applications in various industries, such as packaging, textiles, biomedical implants, 3D printing, and disposable tableware, depending on its crystalline structure (higher crystallinity results in a harder product) [4].

PHAs are a family of thermoplastic polyesters synthesized by microbial fermentation of renewable carbon sources like sugars or lipids [5]. According to their composition and structure, they can exhibit a wide range of properties and applications, such as in packaging, agriculture, and medical devices, in both flexible and rigid forms [6]. Among the different polymers, polyhydroxybutyrate (PHB) has provided the most interesting results. Meanwhile, copolymerization with hydroxyvalerate (HV) can yield poly(-3-hydroxybutyrate-co-3-hydroxyvalerate) (PHBV) with enhanced processability.

PBS is a biodegradable polyester produced from succinic acid and 1,4-butanediol, both of which can be derived from renewable resources. It possesses properties similar to those of traditional petroleum-based plastics like polyethylene (PE) and polypropylene (PP), which enable it to find applications in packaging, disposable items, and agriculture [7].

PCL is a biodegradable polyester synthesized through the ring-opening polymerization of ε-caprolactone. It has a low melting point, making it suitable for applications requiring melt processing, such as 3D printing and drug delivery systems [8].

Similar to PCL, PBAT is a biodegradable polymer synthesized from non-renewable fossil-based sources. It is generated as a result of butanediol and terephthalic and adipic acid polycondensation. Although these components are being recently produced from renewable feedstocks, completely bio-based PBAT is not commercially available [9]. Other related bio-based alternatives have also arisen, such as PBSeT (poly (butylene sebacete-co-terephthalate) [10]. As an aromatic–aliphatic polyester, PBAT shows better processability and mechanical properties than PHB and PCL (aliphatic polyesters) [11], and thus it is used for packaging, mulch films, and, especially, light shopping plastic bags [12]. In Europe, PBAT can be found in commercial products under the name of Ecoflex^®^ and Origo-Bi^®^ or in Mater-Bi^®^, blended with other polymers [13].

Cellulose-based plastics are derived from the modification of cellulose (the most abundant organic polymer) through various methods, including chemical modification, dissolution, and regeneration of cellulose fibers. One example is cellulose acetate, an ester of cellulose produced by the reaction of cellulose with acetic anhydride and acetic acid in the presence of sulfuric acid [14]. According to the number of hydrogen atoms replaced with an acetyl group (called degree of substitution), cellulose acetate can have different forms, but the most common one (known as cellulose acetate) has an acetate group of approximately 2–2.5 of every three hydroxyls [15]. Cellulose-based plastics are fully adopted for disposable packaging, while cellulose acetate still has a limited market (particularly in cigarette filters and photographic films) [16].

Starch is a common polysaccharide consisting of branched amylopectin and linear amylose chains, both of which are made up of glucose molecules. If exposed to heat with the addition of a plasticizer, hydrogen bonds between the starch molecules could be replaced by the plasticizer (water or glycerol), enabling the polymer chain to move more freely [17]. This material is called thermoplastic starch, which is one of the main components of starch-based products, such as plastic films, bags, and agricultural mulching films [18]. However, thermoplastic starch is often used in a blend with other bioplastics such as PLA, PHB, and natural fibers in order to improve the mechanical characteristics of the plastic [19].

The main advantage of these bioplastics is their capacity to biodegrade, which holds significant potential for mitigating plastic pollution [20]. However, biodegradation occurs in different forms, depending on the environment in which it takes place, especially if the environment is industrialized or natural. In addition, although all of these bioplastics show a biodegradation trend, their biodegradability could differ significantly between them, which can have an important effect on the choice of further production stages [21]. The present state of the art includes various studies on bioplastic degradability in different environments, but these results are not usually focused on actual biodegradability; instead, weight loss or visual disappearance are used as indicators [22]. Vardar et al. detailed and compared the biodegradability of different bioplastics, focusing only on anaerobic digestion [23], while Bher et al. considered only the mesophilic temperature range [24]. Another issue is that studies are limited to only a few bioplastics or those most common on the market (PLA, starch-based, or PHA) [25,26], where less common bioplastics such as PBAT, PCL, and PBS remain poorly studied. Indeed, considering the case of PBAT, this bioplastic is unexplored in some of the latest reviews in the literature [27].

In general, there is a lack of detailed assessments of plastic biodegradability in both industrial and natural environments. As a consequence, the present work aims to review the current state of biodegradability of several bioplastics in anaerobic digestion and composting, as well as soil, marine, and freshwater. After presenting the biodegradation mechanisms, the different standard methods are introduced and discussed, followed by a comprehensive assessment of the biodegradability of different bioplastics and their respective labels and certifications.

## 2. Biodegradation Mechanism

There are two main ways in which polymer breakdown can occur: biotic and abiotic. Abiotic degradation, also known as deterioration, occurs via hydrolysis and photodegradation and results in a physical breakdown process that can lead to partial or complete destruction [28]. The association of naturally occurring microorganisms such as bacteria, fungi, and algae is known as the biotic pathway [29]. The biodegradation process is a combination of abiotic and biotic processes (one mechanism being more dominant than the other), where an initial fragmentation by the abiotic process is followed by the biological action of microorganisms and enzymes occurring during the biotic stage. At the end of this process, a significant alteration of the polymer properties like mechanical, optical, discoloration, and phase separation or delamination, among others, takes place [30].

Biodegradation can be further classified into aerobic and anaerobic according to the presence or absence of oxygen.

Aerobic biodegradation is the mineralization of organic compounds by the action of aerobic microorganisms in the presence of oxygen:

Polymer + O_2_ → CO_2_ + H_2_O + NO_2_ + SO_2_ + biomass + heat (1)

Anaerobic biodegradation is the same process but in the absence of oxygen, resulting in the production of methane and carbon dioxide:

Polymer → CO_2_ + CH_4_ + NH_3_ + H_2_O + H_2_S + biomass + heat (2)

The energy stored in organic matter is released in the form of heat. In particular, in anaerobic degradation, this energy is mainly released as methane, and less heat is produced [31].

Because of plastics’ lack of water solubility and the size of the polymer molecules, microbes are unable to carry the polymeric material into the cells, where the majority of biological processes occur, and must instead secrete extracellular enzymes that can depolymerize the polymers outside of the cells [32]. Plastic biodegradation typically occurs due to surface erosion, whereby extracellular enzymes operate only on the polymer surface due to their size, which prevents them from penetrating deeply into the polymer matrix [33]. The mechanism for the biodegradation of plastics primarily consists of the following steps [34]:

- *Microorganism interaction with the polymer surface*: The first phase involves the attachment of microorganisms to the polymer surface, resulting in the formation of a biofilm. When microorganisms infiltrate the amorphous region of the polymer, the primary polymer chain is broken down into low molecular weight oligomers, dimers, and monomers through the action of the extracellular enzymes released by microorganisms (lipase, proteinase K, andhydrogenase, etc.). The polymer porosity is a crucial factor for the duration of this stage.
- *Microorganisms’ growth*: After adhering to the polymer, the microbes multiply by breaking down the polymers and lowering their molecular weight. Another name for this procedure is “biofragmentation”.
- *Ultimate mineralization of the polymer*: Finally, if the plastic’s molecular weight has been sufficiently lowered to produce water-soluble intermediates, these products can be introduced into the microorganisms and find a conduit into the relevant metabolic pathways (bio-assimilation). Only when these low molecular weight polymers are subsequently utilized by microorganisms as carbon sources does the polymer ultimately degrade. The production of metabolites, such as CO_2_, H_2_O, or CH_4_, is referred to as mineralization. After that, these products are used as energy and carbon sources. When either the oligomers or monomers are no longer present, it is considered that the biodegradation process has finished.

Polymer biodegradation is influenced by the combination of environmental factors (humidity, temperature, light, pH, nutrient supply, etc.) and the physio-chemical characteristics of the polymer (molecular weight, morphology, porosity, hydrophobicity, crystallinity, etc.) [35].

Generally, polymers with a shorter chain, more amorphous parts, and less complex formulas are more susceptible to biodegradation by microorganisms [36]. In particular, polymer crystallinity can have a significant influence on the process: a lower crystallinity degree implies the presence of less organized and packed amorphous domains, which are more easily attacked by microorganisms [37]. Moreover, the environment in which the polymers are placed or disposed of play a key role in their biodegradation. The pH, temperature, moisture, and oxygen content are among the most significant environmental factors that must be considered in the biodegradation of polymers since they affect microbial availability, changing the active sites of the enzymes and altering the overall biodegradation rate [38]. It is important to note that the shape of a product does not affect the final degree of biodegradation, but only the speed of the process. In fact, in stiffer samples, the bio-accessibility of the product by degrading microorganisms is limited. Conversely, in more flexible samples or in powder form, biodegradation occurs more rapidly [39]. Indeed, although Funabashi et al. reported that the final biodegradability of PLA powder and flakes from PLA cups achieved similar values (around 80%), the time required to reach this level was about 30 and 100 days, respectively [40].

## 3. Biodegradability Standards

In order to cover the different environments where biodegradation can occur, several standards have been developed over time to evaluate the biodegradation of plastics under different conditions. Organizations recognized at the international level, such as the American Society for Testing and Materials (ASTM), International Organization for Standardization (ISO), and European Standards (EN), work to provide standards that specify (i) the product requirements or (ii) the methods and procedures for the correct execution of the biodegradability test. In general, even if the standards are developed by different organizations, they all provide the test procedures and conditions depending on the specific environment that needs to be replicated (Table 1). Following these standards, plastic biodegradability is assessed under optimal conditions.

Composting conditions are simulated according to the ISO 14855 and ASTM D5338 standards [41,42,43]. In these standards, plastic samples (in the form of pellet, film, or powder) are mixed with an inoculum (mature compost) and an inert material (sand or vermiculite) in an environment wherein temperature, aeration, and humidity are closely monitored and controlled. The main differences in the conditions required by these standards to carry out the tests involve the duration and scale of the simulation and the composition of the matrix to which the test material is exposed. In particular, the ISO 14855 standard is divided into a first part (ISO 14855-1), describing the general method of analysis of evolved carbon dioxide [41], and a second part (ISO 14855-2), which focuses on a specific method based on gravimetric measurement of carbon dioxide (an absorption column filled with soda lime and soda talc) [42]. For the latter method, the ratio between the amount of test sample to add in the reactor and the compost is 1:10.

In general, ISO 17556 provides information on the biodegradation in soil for plastic materials [44], while EN 17033 is specific for plastic mulch films manufactured from thermoplastic materials [45]. As expected, the temperature adopted in ISO 17556 is lower than that in composting, while the duration of the test is extended to 12 months. Moreover, it is important to maintain a soil humidity of 50–60% for the optimal development of aerobic biological processes.

Regarding aerobic biodegradability, the ISO 14851/14852 and ASTM D6691 standards are used to assess aerobic biodegradability in an aqueous medium and marine water, respectively [46,47,48]. These standards are developed in natural or artificial media (fresh or marine water with specific salts, nutrients, and microorganisms) as inoculum, exposing the plastic samples and measuring biodegradation using a carbon dioxide respirometer or an equivalent measurement method.

One difference between the standards is how the inoculation takes place: for aquatic media, sludge from a wastewater treatment plant (with a suspended solid concentration of 30–1000 mg/L) or a suspension of soil/compost (1–5% *v/v*) is used as inoculum and added to the medium. In the marine environment, the media are inoculated by adding a suspension of various isolated marine microorganisms (types and concentrations are listed in the norm) or using a natural sea water sample (where inorganic nutrients are added).

In particular, for the marine environment, ASTM has proposed a specific standard (ASTM D7473/D7473M) to assess the weight attrition of non-floating plastic materials based on sample weight loss over time (the maximum duration is 180 days) [49]. Since the purpose is not to demonstrate ultimate biodegradation, the process is considered completed if the materials achieve at least 30% biodegradability, according to the ASTM D6691 standard.

All these standards provide a percentage value of material biodegradation through the measurement of the organic carbon transformed to gaseous carbon dioxide, used as an index of microbial assimilation and organic fraction mineralization. The carbon dioxide produced by the sample is calculated by subtracting the background CO_2_ production from a reactor (blank) containing only the media in which the biodegradation is occurring (compost, soil, water, etc.). At the same time, the CO_2_ produced by the sample during biodegradation is compared with a positive material, generally cellulose, for which biodegradability has already been assessed.

Anaerobic biodegradability finds application in anaerobic digestion and landfill environments. In ISO 14853, the temperature required in simulated anaerobic digestion is mesophilic [50], while ASTM D5511 and ISO 15985 imply thermophilic conditions for the test [51,52]. Another important difference is the solid content in the reactor: the process can occur at solid (>20% of total solids) or liquid state (<15% of total solids). The inoculum used in this case is a digestate derived by an anaerobic digester working on the same temperature regime and the solid content for which the test is designed. Focusing on landfills, the ASTM has recently proposed a new standard (D7475) to assess the change from aerobic to anaerobic environments over time as landfill depth increases [53]. However, degradation in aerobic environments is indicated by physical changes (loss of tensile strength, molecular weight, possibly resulting in disintegration and fragmentation) and not by biological processes. Indeed, in anaerobic digestion, the degree of biodegradation is measured considering the carbon-based gas present in the biogas, such as CO_2_ (also produced in an aerobic environment) and CH_4_ (present only during anaerobic processes). Even in this standard, the sample biodegradability is assessed from the difference between biogas production in the reactor that contains the bioplastic sample and the same production in the blank, comparing it with the value from the positive material (cellulose).

The process of bioplastic biodegradation has CO_2_ (and CH_4_ in an anaerobic environment) as the main output of the carbon transformation from the polymer. However, before this stage, bioplastics are subjected to other degradation and deterioration processes (derived from microbial activity as well), such as fragmentation and disintegration. These processes cannot be considered actual biodegradation but are crucial phenomena to let further biodegradation start. Such physical degradation, known as disintegration, is standardized by specific standards. The ISO and EN propose standards for laboratory (ISO 20200 and EN 14806 [54,55]) and pilot (ISO 16929 and EN 14045 [56,57]) scales during composting (there are no specific norms for disintegration in different environments). Laboratory and pilot scales differ based on the volume and the temperature adopted.

**Table 1:** Standard test methods to assess plastic biodegradability in different environments and their respective requirements.

Standard	Environment	Volume of the Reactor	Temperature	Duration	Sample/Media	Validity Criteria
ASTM D5338 [43]	Compost	2–5 L	58 °C	≥45 d	1/6	>70% biodegradation of the reference material after 45 days <20% deviation of the percentage of biodegradation within the reference replicates at the end of the test
ISO 14855-1 [41]	Compost	>2 L	58 °C	≤6 m	1/6	>70% biodegradation of the reference material after 45 days <20% deviation of the percentage of biodegradation within the reference replicates at the end of the test 50 < mg CO_2_/g VS < 150 after 10 days for the blank
ISO 14855-2 [42]	Compost	>500 mL	58 °C	≤6 m	1/10	>70% biodegradation of the reference material after 45 days <20% deviation of the percentage of biodegradation within the reference replicates at the end of the test 50 < mg CO_2_/g VS < 150 after 10 days for the blank
ISO 17556 [44]	Soil		20–28 °C	≤24 m	100–300 mg/100–330 g	>60% biodegradation of the reference material at the end of the test <20% deviation of the percentage of biodegradation within the blank replicates at the end of the test
ASTM D5988 [58]	Soil	2–4 L	20–28 °C		200–1000 mg/500 g *	>70% biodegradation of the reference material after 6 months <20% deviation of the amount of CO_2_ (or BOD) within the blanks at the end of the test
ISO 14851 [46]	Water		20–25 °C	≤6 m	100–2000 mg/L *	>60% biodegradation of the reference material at the end of the test
ISO 14852 [47]	Water		20–25 °C	≤6 m	100–2000 mg/L *	>60% biodegradation of the reference material at the end of the test <20% deviation of the amount of CO_2_ within the blank and test replicates at the end of the test
ASTM D6691 [48]	Marine	125 mL	30 °C	≤90 d	20 mg	>70% biodegradation of the reference material at the end of the test
ASTM D5511 [51]	Landfill and anaerobic digestion		52 °C	≥15 d	15–100 g/1000 g **	>70% biodegradation of the reference material after 15 days <20% deviation of the percentage of biodegradation within the reference replicates
ISO 15985 [52]	Landfill and anaerobic digestion	>750 mL	52 °C		>20 g	>70% biodegradation of the reference material after 15 days <20% deviation of the percentage of biodegradation within the reference replicates
ISO 14853 [50]	Anaerobic digestion	0.1–1 L	35 °C	≤90 d	20–200 mg/L *	> 70% biodegradation of the reference material after 60 days 6 < pH < 8 <20% deviation of the percentage of biodegradation within the reference replicates
ISO 13975 [59]	Anaerobic digestion	>1.5 L	55–35 °C	≤90 d	7–10 g/L **	>70% biodegradation reference after 15 days <20% deviation of the percentage of biodegradation within the reference replicates at the end of the test

* of organic carbon; ** of volatile solid; VS = Volatile solids.

Biodegradation and disintegration, being complementary processes, are considered under a specific property called compostability. Compostable bioplastics are biologically decomposed during a composting process at a rate similar to that of other compostable materials and without leaving visible toxic remainders [60]. Compostability, too, is standardized by EN (13432 and 14995 for plastic packaging and items, respectively [61,62]) and the ASTM (D6400) [63]. According to EN, compostability requires the following conditions:

- Chemical test: identification of all constituents, organic matter content expressed by volatile solids (minimum 50%), and threshold values for heavy metals (tabulated values);
- Biodegradability: at least 90% of the organic material is converted into CO_2_ within 6 months;
- Disintegration: after 3 months of composting and subsequent sifting through a 2 mm sieve, no more than 10% residue may remain compared to the original mass.
- Compost quality: final compost quality should not be negatively influenced by the addition of a biodegradable plastic into the original substrate that is to be composted.

Apart from biodegradation and disintegration, compostability involves the ecotoxicity assessment of the compost produced during the previous tests according to the OECD Guideline for Testing of Chemicals 208, Terrestrial Plants, Growth Test [64]. The compost obtained at the end of the composting trial, eventually containing undegraded residuals from the product, should not affect the germination and growth of plants. Ecotoxicity is evaluated by comparing the compost obtained from the bioplastic biodegradation with a blank sample. The germination rate and plant biomass of the sample composts shall be no less than 90% of that of the corresponding blank compost for two different plant species.

Certification for biodegradation per se does not exist and does not have any significance since biodegradation must always be associated with a specific environment such as home and industrial composting, soil, and water [65]. To aid certification and avoid confusion, several eco-labels have been released worldwide, as detailed in Table 2. These labels are released by different certification bodies that examine the compliance of such products with standard methods [66]. The aim of certification bodies is to assess the intricate information mandated by these standards and to impartially and objectively evaluate the overall characteristics of a specific material. This assessment is conducted through certification systems: standards establish the theoretical framework, while certification systems put this theory into practical application.

## 4. Biodegradability in Managed Environments

**Figure 1:**
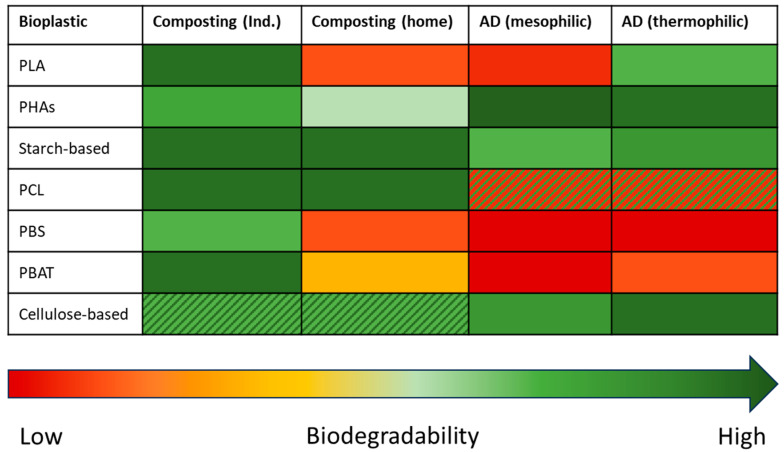
Qualitative value of biodegradability of different bioplastics in industrial environments: aerobic composting (industrial and home) and anaerobic digestion (mesophilic and thermophilic). Adapted from [97].

This section presents the biodegradability of several bioplastics in so-called managed or industrial environments, such as composting and anaerobic digestion. In particular, in order to take into account the effect of temperature, these environments will be distinguished between thermophilic and mesophilic conditions (Figure 1). It is important to note that the results shown in this section refer to the standard methods discussed in the previous section.

PLA degradability has been widely investigated in both composting and anaerobic digestion. Regardless of the type of environment (aerobic or anaerobic), PLA can be rapidly degraded under thermophilic (>58 °C) conditions but slowly at mesophilic temperature: it is necessary to reach the polymer glass transition temperature to increase bioplastic hydrophilicity [70]. In industrial composting adopting thermophilic temperatures, PLA biodegradation can range between 70% and 85% over a period of 4 weeks to 3 months [36]. Having a lower temperature (about 30 °C), the biodegradation level is significantly low (about 30%), and the process takes place over a longer period [71]. In anaerobic digestion, the same trend is maintained, but reaches an overall lower biodegradation level. Degradation between 20% and 40% has been reported for mesophilic anaerobic digestion of PLA after 200–300 days [72], but mineralization of about 1% to 7% has been observed in several studies [73]. At 55 °C, 68% of biodegradation was obtained in 60 days [74], but a higher temperature (80 °C) could accelerate PLA biodegradation to 65% in 3 days [75]. In general, reported times of degradation for PLA are considerably long; therefore, considerably prolonged times are needed for PLA to biodegrade in industrial plants, which always maintain high temperatures.

In the case of PHAs, the behavior in aerobic and anaerobic environments presents some differences. Aerobic biodegradation is strongly affected by temperature: at home or in a curing (mesophilic) compost (where lower temperatures occur), PHAs show reduced biodegradation [76]. On the other hand, biodegradation is improved by higher temperatures, such as those in industrial composting. Studies have revealed how PHA-based bioplastic can biodegrade at 80% after one month, but generally PHA biodegradation occurs at slower speeds [25]. PHBs show full biodegradation after 100 days, but shorter biodegradation can be obtained for PHBV, especially in samples with higher HV concentrations [77]. PHAs degrade significantly better in anaerobic digestion systems than aerobic ones. Under mesophilic conditions, PHAs can fully degrade in a few weeks: 93% of PHB biodegradation in 7 days was reported, while PHBVs showed a slightly slower biodegradation under such conditions than PHB [23]. Under thermophilic conditions, PHA biodegradation can achieve a biodegradation that ranges from 25% to 70% after 11–33 days. Such values, if compared to the ones obtained under mesophilic conditions, are much lower (even less than the half) [23].

A wide range of degradation rates have been reported for starch-based bioplastics in the literature, corresponding to different formulations available on the market. Depending on the application of the product, various polymers can be combined with starch. Generally, starch is dominant in such blends, but examples where it is not are also described. In composting, such bioplastics can achieve a full degradation within 3 months [22], but some examples where lower biodegradation levels occur are present in the literature [78]. In an anaerobic environment, mesophilic temperature offers a biodegradation level of 20–30% after 2–4 weeks [79], but lower biodegradation rates (about 10%) have also been reported. In this context, other polymers can be inhibitory or can enhance the biodegradation of the blend. Under thermophilic conditions, slightly higher degradation rates (nearly 30–50%) of starch-based bioplastics were observed: bags achieved a biodegradation degree of 85% in 23 days or 92% in 35 days [80,81].

During industrial composting, PBS can suitably biodegrade after about 100 days [82], even though studies have revealed how more than 70% of biodegradation can be obtained by adding specific fillers [83]. On the contrary, PBS does not show any anaerobic biodegradability (regardless of the temperature regime adopted): after more than 100 days, slight (2%) or no biodegradation was detected [84]. This trend does not change even by adding biodegradable fillers in the polymer matrix to increase the bio-accessibility of microorganisms [85].

PCL biodegradability has not been widely explored. In composting, PCL has achieved high and fast biodegradability, even at ambient temperature [86]. The anaerobic biodegradability of this polyester has been controversial: Kale et al. [87] and García-Depraect et al. [88] reported that PCL is not anaerobically biodegradable, while Abou-Zeid et al. [89] and Yagi et al. [90] found that the degradability of PCL is possible at mesophilic and thermophilic temperatures, respectively (especially for the latter condition). In general, it is possible to conclude that PCL biodegradation is slower than that of other bioplastics such as PHB and PHBV [89].

In managed environments, the biodegradability of PBAT is well proven. Under composting conditions, high biodegradability is achieved at thermophilic temperature, while mesophilic temperatures do not ensure complete biodegradability in the time required for compostability. Biodegradation of PBAT film after 45 days ranges from 67% to 34% depending on the compost source (manure compost, food compost, and yard compost) [91]. When lower temperatures are adopted, PBAT results in lower biodegradation (<10%) [39]. Regarding anaerobic digestion, thermophilic conditions allow PBAT to reach higher biodegradation levels than at lower temperatures, despite it being limited [92]. Under mesophilic anaerobic conditions (37 °C), the PBAT biodegradation after 126 days was only 2.2%, while under thermophilic anaerobic conditions (55 °C), biodegradation degree was 8.3% after the same time [93].

In composting, cellulose is considered relatively fast-degrading and is used as a reference during laboratory tests. On the other hand, cellulose-based bioplastics are characterized by slow aerobic biodegradation under industrial composting conditions: cellulose acetate fully biodegrades within 200 days [94], while in industrial plants, disintegration is limited to the fillers used [95]. Under anaerobic conditions, materials made of cellulose degrade under both mesophilic and thermophilic temperatures over 60%, with degradation rates more than 80% under thermophilic conditions [96]. Bioplastics made of cellulose are characterized by lower biodegradation, as expected from their chemical composition: most anaerobic bacteria are not capable of degrading lignocellulosic materials, or they are able to hydrolyze them at elevated temperatures. However, the addition of specific additives or plasticizers can ensure full anaerobic biodegradation under mesophilic conditions after 32 days [85].

## 5. Biodegradability in Unmanaged Environments

**Figure 2:**
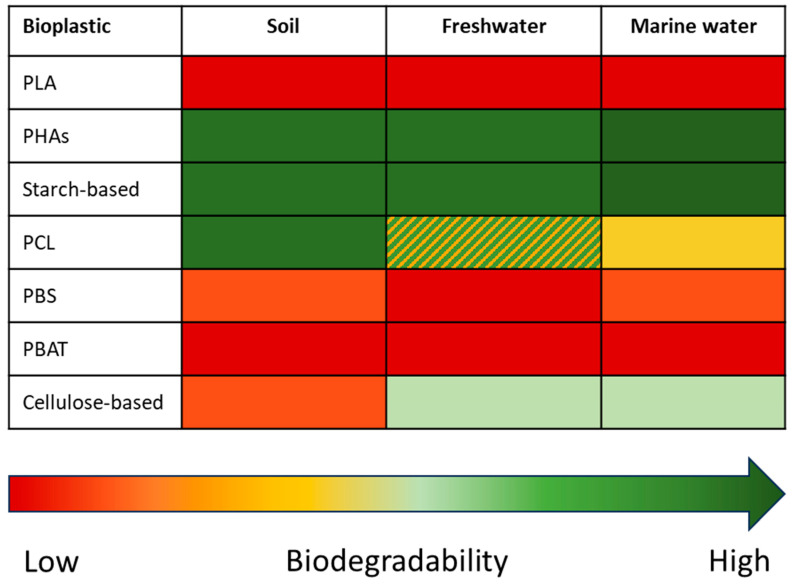
Qualitative value of biodegradability of different bioplastics in natural environments: soil, freshwater, and marine water. Adapted from [97].

This section focuses on biodegradation in unmanaged or natural environments, such as soil, freshwater, and marine water (Figure 2). In those environments, biodegradation is not meant to occur apart for products that have an end of life there (e.g., bioplastic nets or mulching films can biodegrade in an aqueous and a soil environment, respectively). In this context, biodegradable bioplastics, which could have good biodegradability under industrial conditions, can present significant differences in biodegradation in natural environments, since the conditions could be completely different. Thus, the choice of a bioplastic for products related to natural environments is crucial to offering a sustainable and environmentally safe option.

As already introduced in the previous section, the degradation of PLA at mesophilic or lower temperatures is almost negligible. As a result, in soil conditions, PLA does not show any sign of biodegradation or only 4% after 150–200 days [98]. These trends are extended to PLA-based bioplastic blends as well, which do not degrade or show the slowest degradation compared to other blends [99,100]. Apart from slow biodegradability, PLA is not used in soil applications as a mulching film due to its high stiffness. This could be reduced by adding additives and masterbatches, but it is important to consider the effect of these substances on biodegradability and especially ecotoxicity. Similar results have been achieved in aquatic environments (freshwater and marine water) for pure PLA and PLA-based blends as well [97]. If at 50 °C, PLA biodegradation in water occurs after 2.9 months, at 30 or 25 °C, this time increases to 8.5 and 11.4 months, respectively [101].

PHAs have been proven to be biodegradable in natural environments, despite complete biodegradation requiring more time than that necessary under industrial conditions. In a soil environment at 25 °C, PHA powder and PHB film resulted in 95% biodegradation after 150 [102] and 360 days [103], respectively. In an aquatic environment, PHB degraded completely in marine water (30 °C) and freshwater (21 °C) in 43 and 56 days, respectively [97]. PHA films were completely mineralized after 100 days in seawater [104]. PHBV films have been found to be fully biodegradable in marine water in a range between 115 and 250 days [105,106], and they are likely to degrade in freshwater since 53% of mineralization was obtained after only 2 weeks [107].

As well as PHAs, starch-based bioplastics also resulted in complete biodegradation in natural environments [108]: thermoplastic starch was completely biodegraded after 136, 28, and 28 days in soil, freshwater, and marine water, respectively [97]. In particular, this material can achieve more than 60% biodegradation in soil after only one month [109], while a blend of starch-based bioplastic and biodegradable polyester presented a biodegradation phase plateauing after 150 days [110]. This is crucial since thermoplastic starch has poor physical properties for functional use as mulch films, and it is therefore usually blended with other polymers (PCL, PBAT, PBS, or PHA) to improve flexibility, strength, tear resistance, durability, etc. [111]. In freshwater, thermoplastic starch achieved a biodegradation of 75% after 17 days [112], while several thermoplastic starches blended with inorganic fillers reached a biodegradation range of 72–89% after 75 days of testing. The addition of filler to the blend reduced the biodegradability of pure thermoplastic starch [113].

PCL was fully biodegradable under soil conditions after 270 days at 22 °C [114]. While PCL showed excellent biodegradation in terrestrial media, lower values were obtained in aquatic environments. In particular, marine water was found to be more effective for PCL biodegradation (where a value of about 80% was achieved after 3 months [115]) than freshwater environment (about 30% biodegradation after 140 days [116]). However, in some works, full biodegradation was achieved in freshwater after 40 days of testing [117], or more than 80% was reached after 28 days [118].

PBAT exhibits more resistance to microbial action in natural environments and aquatic exposures. For instance, Kim et al. studied PBAT degradation in freshwater, seawater, and wastewater for 30 days, reaching medium biodegradability (32%) only in the latter and almost none in the other two media [119]. In soil, biodegradation presents a similar trend [120]: after 33 weeks of testing, only 2.3% of PBAT biodegradation was achieved [121].

As expected from the aerobic biodegradability values obtained under industrial conditions, where PBS achieved the lowest biodegradability in industrial composting and did not fulfill the biodegradability criteria for home composting, it is reasonable to assume that this bioplastic shows poor biodegradation in natural environments. However, only a few studies have investigated PBS degradability under such conditions using respirometric tests. It has been found that 90% of PBS biodegradation in soil takes place after 440 days [122]. This result is in line with the findings of Narancic et al. [97] and García-Depraect et al. [88], where no sign of biodegradation was detected in soil and freshwater environments, while marine biodegradability was about 20%. Negligible biodegradation in aquatic environments, both freshwater and marine water, has been found in short [123] or long-term assessments [124,125]. Nevertheless, there are some other PBS grades containing some butylene adipate moieties (e.g., poly(butylene succinate-co-adipate) or PBSA) that have demonstrated complete biodegradation in soil, in line with the general finding that the biodegradation rate of PBS increases with increasing adipate-to-succinate ratio [126,127]. In general, PBS results in the slowest biodegradation in the natural environment, considering the limited number of studies available in the literature [128].

Finally, regarding cellulose-based bioplastics, there is a general lack of biodegradability studies through respirometric systems in environmental conditions (soil, freshwater, and marine water) [129]. In principle, cellulose acetate is biodegradable, although degradation proceeds extremely slowly [130]. In soil, cellulose acetate (either in filter or plastic form) degraded slowly with only 5–10% weight loss after 157 days [131], while paper pulp biodegraded by about 30% after 660 days [132]. Cellulose acetate showed approximately 30% weight loss after 35 days of soil burial, but no significant further increase was observed up to the end of the testing period of 120 days [133]. Cellulose diacetate proved to be biodegradable in freshwater and marine environments after 120 and 240 days, respectively, showing an inconstant degradation rate with an important lag phase (75 days) [134]. However, biodegradation in aquatic media appears to be slower than that obtained for other bioplastics [135,136].

## 6. Challenges and Future Perspectives

The message of this work is that bioplastics do not generally degrade similarly in all environments. This has an important implication, for example, in the current compostability standard, which require only aerobic degradability tests, while it is generally not necessary to test biodegradability under anaerobic conditions [96].

Another issue is the discrepancy between the standard presented in the previous section and the actual conditions that occur in industrial and natural environments. Taking as an example the biodegradation standard under industrial composting conditions, it is clear how the temperature and test duration adopted at laboratory scale are not in line with those occurring in a composting plant: the composting treatment reaches thermophilic temperature only for one month, while in the standard, thermophilic temperature is maintained for the whole duration of the test. Similarly, the standard conditions referred to as environmental environments do not accurately reflect actual conditions: in the EU, average marine and freshwater temperatures are 9 and 12 °C, respectively, with a huge variability between the environment under investigation [137]. For all these reasons, a continuous update of standards is crucial for a viable end-of-life of bioplastics.

In addition, it is important to note that bioplastic biodegradation usually occurs in media where different substrates coexist. In particular, if these substrates are more easily degradable (e.g., food waste), bioplastics could exhibit even lower biodegradation in the same environment, temperature, and treatment time. Microorganisms focus their degradation on these compounds, attacking bioplastics with lower effectiveness [138]. Parameters such as speed and degree of bioplastic degradation are particularly important for industrial plants, as the organics fed to digesters and bioplastics should degrade at similar rates in order to acquire a clean output.

The limited biodegradability of bioplastics when they reach their end-of-life could be enhanced in several ways. First, specific pre- or post-treatment could be implemented by industrial facilities. Mechanical shredding, alkali addition, thermal oxidation, or UV irradiation (and their combination) have been widely explored for bioplastic pre-treatments but are still not implemented at full scale [139]. On the other hand, organic waste facilities could implement higher temperatures for a longer time or longer treatment time as well to increase bioplastic biodegradability [140] and provide specific sorting and screening systems to remove non-biodegradable bioplastic fragments, which can be recirculated in the treatment chain [141]. Furthermore, the enrichment of specific microbial consortiums with a focus on their activity in biodegrade bioplastics could be a viable option since several microorganisms able to biodegrade bioplastics have been isolated [142].

Another solution is the development of innovative bioplastic blends able to enhance their biodegradability in the specific environment under assessment. Appropriate blending has proven to improve bioplastic properties (such as mechanical strength, ductility, flexibility, etc.) [143,144] and to enhance biodegradation as well [145,146,147]. Indeed, some material used in the blend can be degraded in the first stage, leaving the matrix of the remaining polymer more accessible to microbial attack [148]. However, the choice of the blending materials should be done with care since several cases have been reported where biodegradation has been limited [149,150] and the ecotoxicity in compost or animals has increased [151,152,153]. These reasons highlight the necessity of having proper characterization of materials and additives when blended with biopolymers.

The issue of limited biodegradability under real conditions is exacerbated by limited customer education to handle bioplastics, resulting in mismanagement of these materials. Compostable plastics could be found in regular recycling bins or improperly littered in natural environments, assuming that they will biodegrade in any conditions. The consumers’ difficulties in verifying the nature of the product/packaging purchased represent a barrier to market development. Therefore, even if European consumers perceive bioplastic as an environmentally friendly choice, they do not recognize it as such at the point of purchase. The reason for this confusion could lie in the users’ lack of clarity regarding the terms bio-based and biodegradable terms, as well as eco-labels. Informing citizens about waste separation is challenging but necessary, since they are a fundamental part of bioplastic waste management.

Although economic, technical, and social factors still persist on the bioplastic market and end-of-life [154], these materials can actually contribute to a more sustainable circular bioeconomy. In general, different studies have confirmed the general preference of bioplastics over fossil-based plastics in countries such as the Netherlands, Italy, the USA, and Germany, since they believe that these products have a more positive environmental impact. This drives users to pay more for horticultural items and packaging (10–20% and 6–24% more, respectively) instead of buying fossil-based plastic alternatives, particularly if clear and pro-environmental labels are present on the packaging [155]. The main reason for this choice is the “perception of naturalness” of bio-based plastics (especially for clothes) and the reduction of the dependency on crude oil and of CO_2_ emissions [156]. The adoption of bioplastics is dependent on people’s level of awareness of the environmental impacts of their consumption and their ability to translate their attitude into behavior. When the level of education is high, most people try to avoid any type of plastic packaging (regardless of the biodegradability aspect or the source) [157]. In general, biodegradability seems to be more appreciated than the bio-based source, and more biodegradable plastic items for daily use on the market are welcomed by users [158].

## 7. Conclusions

The plastic industry is evolving toward a more sustainable approach, and one example is the introduction of bioplastics to the market. However, care should be taken when a specific bioplastic is used for a particular application. Indeed, biodegradability is strictly influenced by the environmental conditions present at the end of a product’s life. In general, starch-based bioplastics and PHAs have suitable biodegradability in all the environments under assessment, while PLA could be easily degraded only when thermophilic temperatures are adopted. While it is clear how viable biodegradability occurs only during industrial composting for PBAT and PBS, the same cannot be said for PCL, which should be investigated more deeply (especially for anaerobic digestion). Appropriate labeling should be used to mark the respective trends in biodegradability in industrial and natural environments, ensuring that the public can easily understand such labels. To increase the knowledge and, consequently, the acceptance of bioplastics, a comprehensive political, social, and economic policy should be pursued. The focus should be placed on four main fields: (i) technological, improving the properties and biodegradation of bioplastics themselves; (ii) financial, reducing the costs of bioplastics production and facilitating their competitivity with fossil-based plastics; (iii) waste management, creating specific separate collection streams, waste treatment, and recycling facilities, as well as extended producer responsibility schemes; and (iv) public awareness, facilitated by clearer and more understandable labeling of products.

## Figures and Tables

**Table 2:** Main global bioplastic certification bodies with respective standards and acceptability.

Certifying Body	Country	Label	Standard	Acceptance
DIN Certco	DE	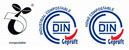	EN 13432 [62] ASTM D6400 [63] EN 14995 [61] ISO 17088 [67]	Europe
		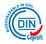	ISO 17556 [44]	
ORG	UK	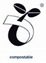	EN 13432	Europe
KEURMERK INSTITUTE	NL	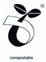	EN 13432	Europe
COBRO	PL	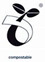	EN 13432	Europe
ABA	AU	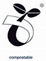	EN 13432	Europe/Australia
TÜV Austria	BE	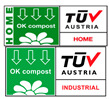	EN 13432	Europe
		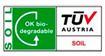	ISO 17556	
		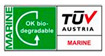	ASTM D6691 [48]	
		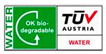	EN 14987 [68]	
Finnish Solid Waste Association (FSWA)	FI	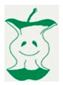	EN 13432	Finland
CIC	IT	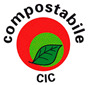	EN 13432	Italy
Avfall Norge	NO	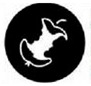	EN 13432	Norway
Generalitat Catalunya	SP	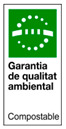	EN 13432	Spain
Biodegradable Products Institute (BPI)/US Composting Council (USCC)	US	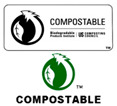	ASTM D6400	USA
BNQ	CA	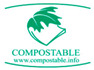	BNQ 9011-911/2007 [69]	Canada
BPS	JP	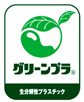	Green Scheme Pla	Japan

## Data Availability

No new data were created or analyzed in this study.
